# Improving the Surface Quality and Tribological Characteristics of 3D-Printed Titanium Parts through Reactive Electro-Spark Deposition

**DOI:** 10.3390/ma17020382

**Published:** 2024-01-12

**Authors:** Georgi Kostadinov, Todor Penyashki, Antonio Nikolov, Aleksandar Vencl

**Affiliations:** 1 Institute of Soil Science Agrotechnologies and Plant Protection “N. Pushkarov”, Agricultural Academy, Shose Bankya Str. 7, 1331 Sofia, Bulgaria; gdkostadinov@gmail.com; 2 Faculty of Industrial Technology, Technical University of Sofia, Kliment Ochridsky 8, 1000 Sofia, Bulgaria; anikolov@tu-sofia.bg; 3 University of Belgrade, Faculty of Mechanical Engineering, Kraljice Marije 16, 11120 Belgrade, Serbia; avencl@mas.bg.ac.rs

**Keywords:** selective laser melting (SLM), Ti6Al4V, roughness, microhardness, wear resistance

## Abstract

This work presents the results of research conducted with an aim to improve the surface quality, hardness and wear resistance of titanium alloy Ti6Al4V, obtained via the laser powder bed fusion of metals (PBF-LB/M) process of additive manufacturing (AM) known as the 3D printing of metals. The 3D surfaces were coated via reactive electrospark deposition (RESD) with low-pulse energy and electrode materials of low-melting metals and multi-component hard alloys. The relationship between the electrical parameters of the RESD process and the quality, composition, structure, microhardness and wear resistance of the treated surfaces were investigated and analysed. It was found that the roughness and thickness of the resulting surface layers could be changed by changing the RESD modes within the limits of 2.5–5 µm and 8–20 µm, respectively. RESD processing allowed us to achieve two to five times lower roughness than that of titanium AM surfaces. The microhardness and wear resistance of the RESD surfaces are two to four times higher than those of the titanium substrate. Possibilities for the purposeful synthesis of new wear-resistant phases and compounds and for obtaining surface layers with predetermined thickness and roughness were established. It was shown that the subsequent reaction’s electrospark processing helped to simultaneously reduce the roughness and increase the hardness and wear resistance of the modified surfaces, and can be successfully used instead of the material-energy-labour and machine-intensive finishing treatments of the titanium surfaces obtained after 3D printing.

## 1. Introduction

Additive manufacturing (AM) technologies or the 3D printing of metals are becoming increasingly popular and allow the creation of the most complex products in terms of shape and configuration, spatial details, and assemblies and structures, without the need for welding and/or assembly and with reduced production costs and a minimisation of the required amount of material [1,2]. Laser powder metal fusion (PBF-LB/M), widely known in the industry as laser metal fusion (LMF) and also as selective laser melting (SLM), or selective laser sintering (SLS) [2] and direct laser metal sintering (DMLS) [3] are the most commonly used methods among the industry’s forms of additive manufacturing (AM). They are the best way to produce titanium and titanium alloy products [4] (hereafter referred to as SLM products in short) since the poor machinability of titanium and the presence of expensive titanium waste are avoided. Additionally, it becomes possible to optimise the product to be lighter and stronger. At the same time, the direct production of details and assemblies is realised only on one machine with a single production process, uniting and harmonising various processing and assembly operations without the need for technical training, tools and technological equipment and without the use of various metalworking machines. However, two main reasons prevent the wider application of AM–PBF-LB/M for the production of titanium products.

The first reason is the low hardness and low tribological characteristics of titanium alloys, which limit the scope of their application. Attempts of researchers to avoid them by creating new alloys or appropriate heat treatment have not yet led to satisfactory results [4,5,6]. Currently, for the improvement of the surface properties of titanium and its alloys, numerous studies have been conducted that are aimed at creating wear-resistant coatings using mainly the methods known and established in practice [6,7,8,9,10,11]. However, the use of the known methods is associated with several limitations and difficulties resulting from their physical nature and technological features. The high prices and complexity of equipment and technologies, unsatisfactory bond strength with the substrate, duration of the processes, annealing, recrystallisation and thermal deformation of the substrate, impossibility of depositing coatings with greater thickness, high costs and environmental pollution are some of the main difficulties preventing the use of many of these methods [12].

The second reason is the high surface roughness and the presence of defects such as micropores and microcracks on the surface of the SLM titanium products, which require complex finishing treatments associated with various machines and technologies and with excessive losses of time, labour and energy [13,14]. Many researchers are working on solving these problems, but so far there is still no method effective enough to reduce roughness and clean the surface defects. For example, laser beam re-passing [15] and laser texturing [16] can solve these problems, but both methods almost double the time and cost of SLM fabrication. The impossibility of good cleaning and a satisfactory reduction in surface roughness is a serious obstacle to the application of this technology for the production of titanium products [17]. Currently, issues of surface quality control of SLM and DMLS products, as well as improving the surface characteristics of titanium and its alloys, are becoming increasingly relevant.

Electrospark deposition (ESD) is a suitable and ecological method for improving the surface quality and surface characteristics of titanium SLM and DMLS products. Along with a possibility of controlled production of coatings with a wide range of compositions, structures, and parameters, compared to existing methods for applying wear-resistant coatings, the main advantages of ESD [18,19] are the high adhesion of coatings to the titanium substrate, the possibility of local surface treatment to apply the coatings on external surfaces of any shape and size in strictly defined locations without the need for means and measures to protect the rest of the surface of the product, the ease of deposition under normal atmospheric conditions, the lack of heating, the thermal impact and deformation of the layered product and its high environmental resistance, the universal, simple, cheap, reliable and accessible technology and equipment, the low material costs, the low energy and labour intensity of the process, the possibility to change the properties of metal surfaces in a wide range, and the possibility of operation without additional mechanical processing. This work aims to study the topography, structure, composition, microhardness and wear resistance of the coatings via the appropriate selection of modes, pulse energy and electrodes for ESD to achieve a reduction in roughness and surface defects and an improvement in the structure and properties of SLM and DMLS titanium surfaces, as well as to evaluate the possibilities of replacing the expensive labour-intensive finishing treatments with the cheap and simple ESD technology. ESD is primarily used to harden metal surfaces by transferring hard and wear-resistant materials mainly from hard-alloy electrodes. According to the results of many researchers [20,21,22], the temperature in the discharge microsection of the electrode surface is 5000–10,000 °C, and the intensity of the electric field is 10^7^–10^9^ W/cm^2^, which is much more than the critical intensity at which the electrodes melt, and the ongoing transfer is in mixed vapour, liquid and solid (from brittle breakage of the electrode) states. At the hard alloy electrodes, the presence of the transferred solid phase is relatively high and at such a rate of mass transfer, the formed coatings usually have increased roughness compared to that of the substrate. To reduce the roughness, in this work, reactive electrospark deposition (RESD) [20,23,24] was carried out. The purpose of RESD is to form a local liquid reaction phase on the surface of the substrate, in which the components of the electrode and the substrate actively mix, fill the voids and pores and distribute evenly on the layered surface, reacting with each other to form new phases.

**Figure 1:**
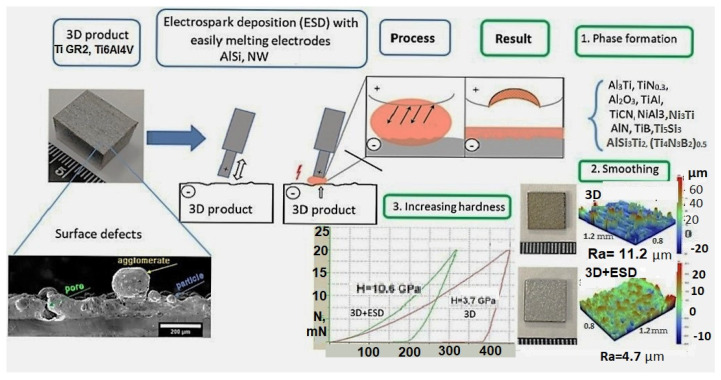
Principle diagram of local melting and formation of a reactional phase and coating after RESD on AM-SLM products.

Signs of active interaction of the electrode and substrate material under conditions of local melting in the ESD process, in which the properties of the substrate are improved as a result of the synthesis of new phases, have been noted in many works [23,24,25,26,27]. In addition, the extremely high cooling rate of the reactive melt (10^5^–10^6^ °C/s) [19,21,22] allows the formation of new ultra-dispersed, amorphous and nanoscale structures with improved wear resistance. The conditions for the formation of the reaction phase and the further development of this technology have not been sufficiently studied. The use of low-melting and eutectic electrode materials is also poorly studied, although it is well known that when the melt solidifies, they form a smooth surface due to their good fluidity and deep supercooling. Based on the available literature data and results of our previous studies [28,29], the necessary conditions for the local melting and formation of a liquid phase on the titanium surfaces were determined, and are shown in the principle diagram of the RESD process (Figure 1).

## 2. Materials and Methods

### 2.1. Substrate and Coating Materials

A titanium alloy, Ti6Al4V (UNS R56400 or ASTM grade 5), designated as Ti-GR5, and commercially pure titanium (UNS R50400 or ASTM grade 2) designated as Ti-GR2 were used as the substrate. The substrates were model square wafers with the dimensions 12 × 12 × 4 mm fabricated via AM (SLM and DMLS) with different mode parameters and different roughness values, Ra = 10–15 μm and Ra = 8–12 μm, respectively.

Low-melting Al–Si alloys with composition and designations AlSi9 and AlSi12 [20,23,24,25] were selected for RESD. The electrodes with a homogenised chemical composition and fine metal–glass structure were created using a special technology developed at the National University of Science and Technology, MISIS—Moscow [20,23]. Hard alloys based on WC-TiB_2_-B_4_C with a low-melting solder multi-metallic mass (Ni-Cr-B-Si-Fe-C) and designations NWW15B10T20 (or NW) [28] were used as a benchmark for a comparison of the characteristics of the coatings from the “AlSi”-type electrodes.

### 2.2. Equipment and Conditions of ESD

It is widely known [18,19,20,21] that the characteristics and properties of electrospark coatings depend mainly on the material of the electrode and the electrical parameters of the deposition mode (current, voltage, capacity, duration and frequency of the pulses, which are most often expressed by the energy of the unit impulse, or impulse energy, *E*). The increase in the values of the electrical parameters of the mode, respective to the pulse energy, causes an increase in the thickness and roughness of the coatings. To obtain smoother surfaces in the present research, equipment with low-pulse energy was used, i.e., *E* = 0.01–0.07 J. Two ESD schemes were used.

**Figure 2:**
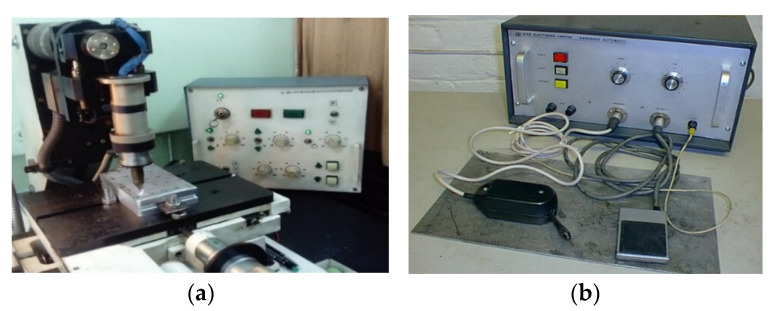
Devices for reactive electrospark deposition: (**a**) LESD equipment and (**b**) ESD with vibrating electrode equipment.

Semi-automatic non-contact local electrospark deposition (LESD) [28] is where deposition is performed with a rotating electrode with the automatic maintenance of the discharge, inter-electrode distance and controllable movement speed of the processed workpiece along the *x*- and *y*-axes (Figure 2a). This ensures the production of coatings with higher density and uniformity, with lower roughness and with very good repeatability of quality characteristics, but also with lower thickness. For LESD regimes, there was a pulse current amplitude of *I* = 16–22.4 A, voltage of *U* = 100 V, pulse duration of *T*_i_ = 8, 12 and 20 μs, capacitance of *C* = 0.68–4.4 μF, pulse frequency of *f* = 5–8 kHz, coating deposition speed of 0.5–1.0 mm/s, number of passes of the electrode of *n* = 2 or 3, rotation speed of the electrode of 1000 rpm and pulse energy of *E* = *U* × *I* × *T*_i_ = 0.02–0.045 J.

Manual ESD via the reciprocating (vibrating) movement of the electrode (Figure 2b) was conducted with the following parameters: a short-circuit current of 0.2–2 A, voltage of *U* = 80 V, capacity of *C* = 3.5–20 µF, pulse energy of *E* = *C* × *U*^2^/2 = 0.01–0.07 J, oscillation frequency of the vibrator of 200 Hz, pulse durability of *T*_i_ = 50 and 100 µs and number of passes of the electrode of *n* = 3.

The work used modes with pre-optimised parameters in terms of the degree of filling of the micro non-uniformities of the SLM surfaces, and the roughness and density of the coatings. The parameters of the used modes given in Table 1 are numbered in the order of the increase in pulse energy. As an indicator for comparing the individual modes, the pulse energy is used.

The coatings were applied with two or three passes of the electrode in the following sequence. The entire surface of the sample was covered with one pass of the electrode, then a second layer was applied over the first layer under the same conditions, and if the initial irregularities were not completely smoothed, a third layer was applied. However, the majority of the coatings were deposited with three passes (n).

### 2.3. Measurement Methodology

Surface topography, microhardness, thickness, composition, structure and wear resistance were used as the main indicators with which to evaluate the characteristics and properties of the RESD titanium surfaces.

The following parameters of surface roughness of the substrates and coatings were measured with profilometer AR-132B (Shenzhen Graigar Technology, Co., Ltd., Shenzhen, China) in two mutually perpendicular directions according to the ISO 21920 standard [30]: arithmetical mean deviation, *Ra*; root mean square deviation, *Rq*; maximum height of profile, *Rt*; and ten-point height, *Rz*. Five parallel measurements in both directions were performed, and the arithmetical means were calculated.

The Vickers microhardness (*HV*) was measured from above (on the top of the coating) after polishing the surface irregularities, with a “Zwick 4350”-(ZwickRoell, GmbH & Co., KG, Ulm, Germany) hardness tester equipped with a Vickers diamond prism indenter at a load of 2 N, at time 10s, and at a penetration depth of 3–4 µm. In order to eliminate the influence of the substrate, the measured hardness was calculated according to the methodology presented in [31]. The number of parallel measurements was 10.

The thickness, δ, was measured with an indicator clock with an accuracy of 0.001 mm. The results are the arithmetic mean of 5 parallel measurements.

The microstructure of the coatings has been studied using a metallographic optical microscope, “Neophot 22”(Carl-Zeiss, Jena, Germany). Phase identification, a determination of the distribution of elements in the surface layer and an additional microstructural analysis of the coatings were performed with a Bruker D8 Advance X-ray diffractometer (Bruker AXS, Karlsruhe, Germany) in “Cu Kά” radiation and with a scanning electron microscope (SEM), “EVO MA 10 Carl Zeiss” (Carl Zeiss, Jena, ZEISS Microscopy, DeutschlandGmbH-Germany). The X-ray energy dispersion EDS microanalysis was performed with an X-ray energy dispersion microanalyser EDX system, “Bruker” (Bruker AXS, Karlsruhe, Germany), and a scanning electron microscope (SEM), “EVO MA10 Carl Zeiss” (Carl Zeiss, Jena, ZEISS Microscopy, DeutschlandGmbH-Germany).

The determination of the coefficient of friction (μ) and tangential force (Ft) was performed with CSM REVETEST Scratch Macrotester (Anton Paar GmbH-Germany) with a Rockwell C diamond indenter. A progressive load mode with a normal force range of 0 to 50 N was used at a speed of 10 N/mm.

Comparative wear tests were performed with a pin-on-disc tribometer under dry sliding conditions with hard-fixed abrasive particles (corundum of grit size P1200) in planar contact between the coated samples (pin) with dimensions of 12 × 12 × 4 mm and abrasive paper under a load of 5 N in a nominal contact area measuring 144 mm^2^, with a nominal contact pressure of 3.47 N/cm^2^ and a sliding speed of 0.239 m/s. The mass loss for a specific sliding distance, under constant conditions of load and sliding speed, was measured. The mass loss was measured continuously, i.e., after 7, 14, 21 and 28 m. The following wear characteristics were calculated: mass loss, wear rate and wear resistance. The results are the arithmetic mean of 3 parallel experiments.

## 3. Results and Discussion

### 3.1. Coating Roughness, Thickness, Structure and Microhardness

Table 2 shows the values of the amplitude parameters of roughness (*Ra*, *Rq*, *Rz* and *Rt*), thickness (*δ*), and microhardness (*HV*) of the surfaces coated under different impulse energies.

**Figure 3:**
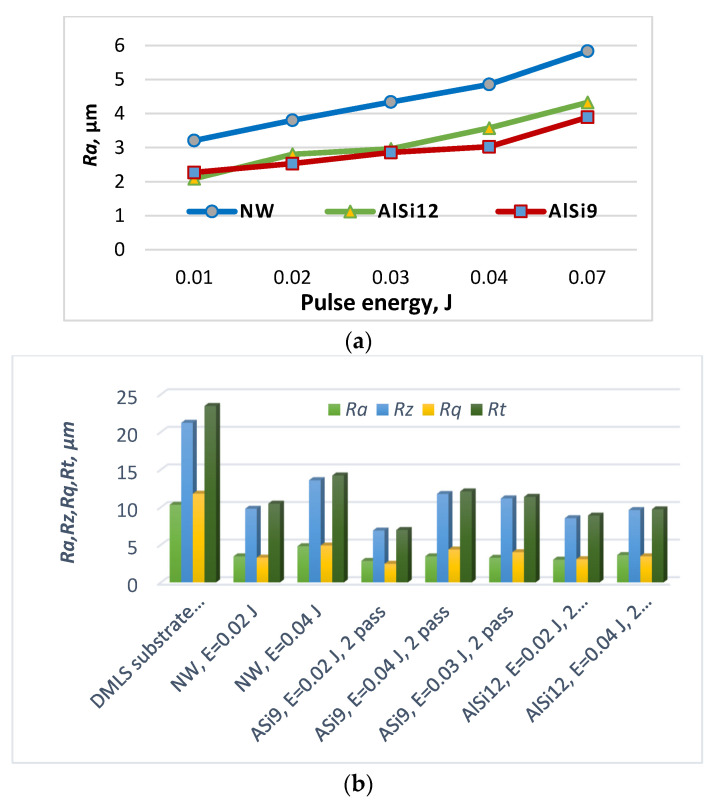
Variation in roughness parameters of DMLS titanium (Ti-GR5) surfaces as a function of pulse energy. (**a**) Surface roughness, Ra, depending on pulse energy in the ESD process with different electrodes. (**b**) Roughness parameters in LESD with NW and AlSi electrodes at 2- and 3-electrode passes.

Figure 3 shows the variation in roughness parameters of RESD surfaces obtained via ESD and LESD on DMLS Ti-GR5 with an initial roughness of *Ra* = 10–12 µm as a function of pulse energy, with other conditions being equal.

It can be seen that the roughness of the coatings from AlSi9 electrodes is lower than that of the substrate and is close to that obtained with ASi12 electrodes. The NW electrode-modified surfaces also show lower roughness parameters than those of SLM and DMLS surfaces, but higher ones than those of AlSi electrodes.

**Figure 4:**
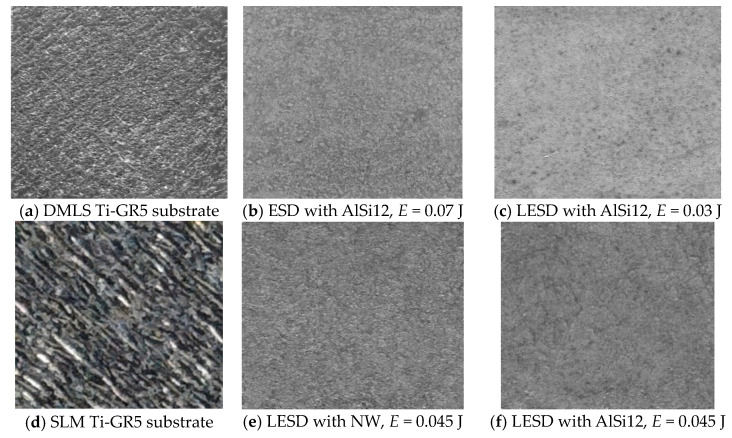
Optical images (20×) of RESD surfaces obtained via ESD and LESD with NW and AlSi12 electrodes on DMLS and SLM titanium GR5 with an initial roughness of *Ra* ≈ 10 µm (**a**–**c**) and *Ra* ≈ 15 µm (**d**–**f**).

**Figure 5:**
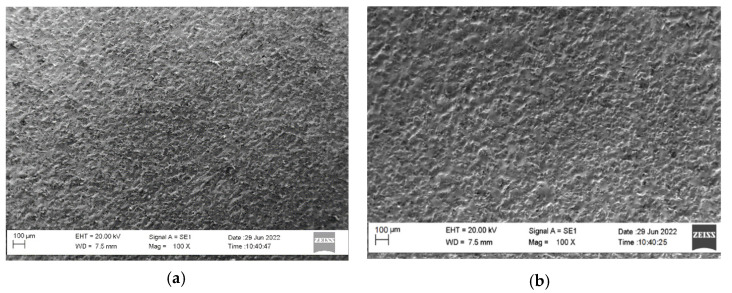

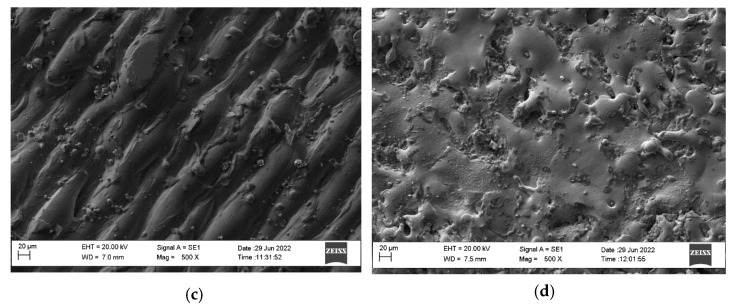
SEM images of SLM Ti-GR5 surface and of RESD-treated surfaces. (**a**) AlSi9/Ti-GR5 ESD, *E* = 0.04 J, *Ra* = 2.5 µm; (**b**) AlSi9/Ti-GR5, ESD, *E* = 0.07 J, *Ra* = 3.5 µm; (**c**) SLM substrate Ti-GR5, *Ra* = 16.5 µm; (**d**) After ESD with NW, *E* = 0.07 J, *Ra* = 3.7 µm.

Apparently, the higher melting temperature of NiCrBSi metal mass and the presence of carbides and borides have led to both the higher roughness, thickness and microhardness of the NW coatings (Table 2). It was found that with an increase in the pulse energy, the roughness, thickness and microhardness of the resulting coatings increase. Therefore, reducing the pulse energy would reduce the roughness and improve the quality of the coatings, but the thickness and microhardness of the coatings would also decrease. The smoothing of the micro uniformities is also confirmed by the general view of the topography of the coatings in Figure 4 and Figure 5, which illustrate characteristic optical and SEM micrographs at different magnifications.

Dense, structurally similar surfaces with a specific relief different from the original one are observed, and are mainly formed by a liquid phase. The liquid phase is in the form of smoothed areas of the surface, formed as a result of the coalescence of the drops from the individual spark discharges. From the comparison of the optical images in Figure 4, which show a larger area of the initial and modified surfaces, it is found that after the RESD treatment, the original roughness of the SLM and DMLS substrates is significantly reduced. It can be seen that the AlSi12 electrode-coated surfaces (Figure 4b,c) are more uniform and smoother than those of the NW electrode (Figure 4f and also that with increasing pulse energy, the roughness and surface irregularities show an increasing trend. At the same time, even at the highest energy used in the work of 0.07 J, the modified surface is smoother and more uniform than that of the titanium substrates.

SEM observations show that the uppermost surface of the coatings (Figure 5a,b,d) can be conditionally divided into three main structural zones, located in different sections of different sizes on the surface of the coating; (i) the glass-like one is the most clearly visible and is predominant in Figure 5d, which shows that it is relatively smooth and homogeneous, and in which the individual structural components are not noticeable. Most likely, these are amorphous phases doped with the metals of the electrode material and saturated with ultradisperse nitrides, carbides and borides from the solid phase of the NW electrode, or obtained as a result of chemical reactions between the elements of the two materials and the environment. Glass-like regions are also seen under ESD with AlSi9 electrodes in Figure 5a,b. It can be noted that at a pulse energy of 0.07 J, their sizes are higher than those obtained at an energy of 0.04 J (Figure 5a). In LESD, the glass-like zone is predominant in the surface of the coatings and the total area of the individual sections is higher than that of coatings obtained via vibrational ESD, which is apparently due to the lower duration of the pulses and the absence of mechanical contact between the electrode and the layered surface; this shows (ii) a crystalline zone that is relatively uniform, but not as smooth as the glass-like one, in which ultra-disperse to small, round-shaped constituents up to a few microns in size are observed (Figure 5a,b). (iii) The area of applied material made of fragile broken particles from the electrode consists of separate single protrusions in the relief of the coatings—“bumps” with an irregular shape of a truncated cone, or a pyramid and sizes reaching, in some cases, up to several microns—most clearly visible in Figure 5b,d. These are individual, larger, softened and partially melted particles of the electrode material transferred and attached to the substrate. When reducing the energy of the pulses, both the quantity and the size of the “bumps” decrease. While the first two structural zones are firmly attached to the substrate, most of the bumps can be removed from the coating, leaving an area resembling the crystalline structural zone underneath. The initial protrusions left after SLM processing (Figure 4a,d and Figure 5c) are melted or burned off as a result of spark plasma discharges.

**Figure 6:**
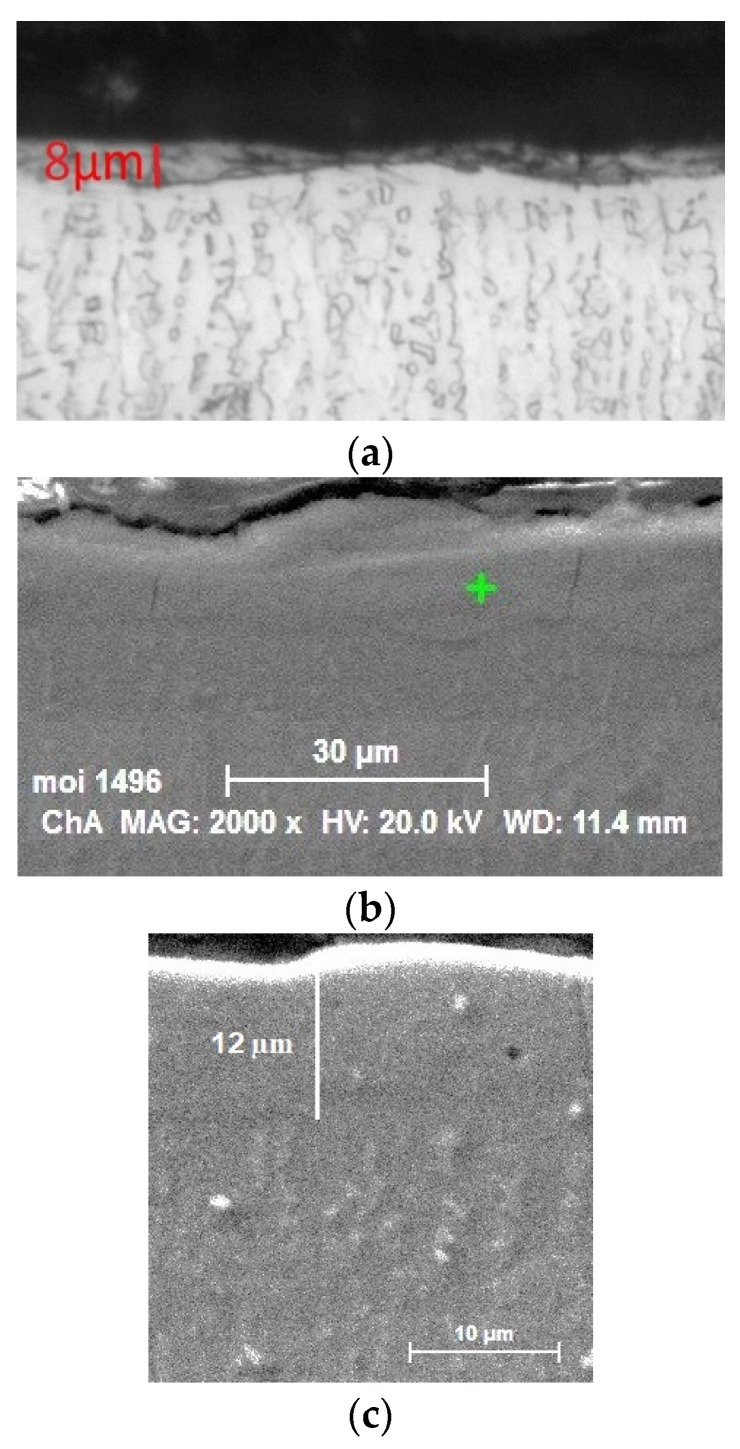
Cross-sections of LESD AlSi coatings on DMLS Ti6Al4V alloy. (**a**) LESD with AlSi12 electrodes, *E* = 0.02 J; (**b**) LESD with AlSi12 electrodes, *E* = 0.04 J; (**c**) LESD with AlSi9 electrodes, *E* = 0.04 J.

Figure 6 shows characteristic cross-sections of the obtained coatings. Melt-filled voids and surface pores are visible, as well as the relatively flat and smooth surface of the coatings.

The microhardness (*HV*) values of the coatings vary over a wide range. For example, the maximum and minimum measured values of the AlSi12 coating, ESD, *E* = 0.04 J (Table 2), were 12.08 and 6.12 GPa, and after discarding the sharply deviating values via the Grubbs method, the mean calculated value was 8.85 GPa. The average calculated values given in Table 2 are 2–3.3 times higher than those of the uncoated substrates. Higher values were obtained for RESD with the NW electrodes. From the data in Table 2, a trend towards a slight increase in microhardness with increasing pulse energy can also be observed.

Analysing the results of the studies of the topography of the coated surfaces shows that dense and commensurable coatings with a given thickness and roughness can be changed by changing the conditions for their application within the limits of *Ra* = 2.5–6 µm, and a corresponding thickness of δ = 6–16 µm was obtained via RESD. The application of RESD via ESD and LESD with pulse energy of up to 0.07 J on the titanium surfaces with an initial roughness of *Ra* > 5–6 μm allowed the simultaneous erasure of the irregularities and traces from previous processing, to fill the pores and the cavities, and to obtain surfaces with improved uniformity and up to four times lower roughness compared to that under SLM and DMLS processing. The highest effect degree of at-once smoothing and erasing of surface defects, filling of pores and cavities and lowest roughness is achieved in RESD with LESD at pulse energy 0.02–0.03 J with AlSi12 electrodes.

### 3.2. Phase Formation, Composition and Structure of Modified Surfaces

**Figure 7:**
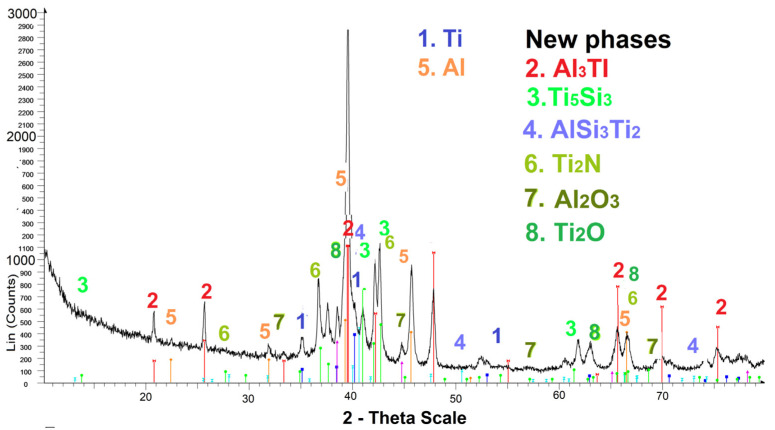
XRD pattern after LESD with AlSi12 electrode on SLM Ti-GR2 substrate, *E* = 0.02 J.

XRD analyses revealed that the composition of the coatings differed from the composition of the starting electrode materials. Figure 7 shows the difractogram of LESD coatings from AlSi12 and NW electrodes.

**Figure 8:**
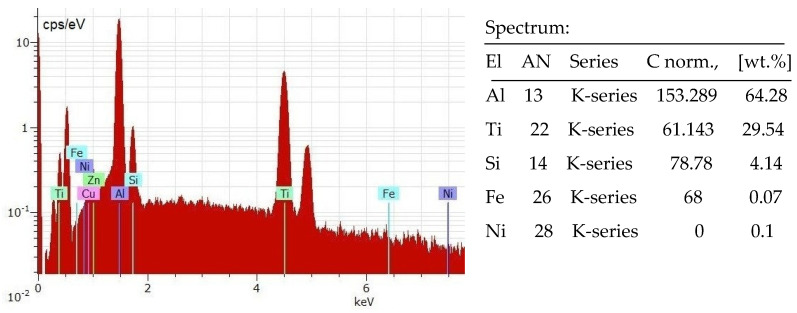
EDS distribution of elements after RESD with AlSi12 electrode on Ti-GR2.

The registered new phases in the compositions of the AlSi coatings are Al_3_Ti and Ti_3_Al, Ti_5_Si_3_, AlSi_3_Ti_2_ and TiN_0.26_. In small quantities, these are registered Al_2_O_3_, AlN, Si_3_N_4_, TiO_2_ and Ti_2_O, which shows that, in the RESD process, titanium and aluminium react with nitrogen and oxygen from the air, forming new wear-resistant oxides, carbides and nitrides. Figure 8 shows the EDS distribution of elements in the AlSi electrode coating, which confirms the presence of the main elements of the registered phases of Figure 7.

The new phases registered in the NW coatings on Ti-GR2 are TiN, WC1-x, TiB, TiC_0_._7_N_0_._3_, WB, Cr_2_B_3_, TiC_1-x_, NiTi_3_, (Ti_4_N_3_B_2_)_0.8_ and Cr_0.2_Ti_0.3_. Also registered are small quantities and traces of wear-resistant compounds and intermetals such as B_4_C, AlN, Si_3_N_4_, CoB, NiB, Ni_3_Ti, CrNiW, Ti_3_Ni and Ti_2_O. It can be seen that the number of phases registered in the coatings is higher than that when using AlSi electrodes.

The registered newly formed phases entail higher microhardness, a stronger connection with the substrate and, accordingly, a higher wear resistance. The broadened characteristic peaks indicate the presence of amorphous structures, which agrees with the data from the SEM images in Figure 5 and also indicates the improved mechanical properties and wear resistance of modified surfaces. However, the presence of peaks suggests also the presence of a crystalline structure, which can be a sign of the formation of a mixed amorphous crystalline structure.

The LESD process appears to be more favourable for the formation of amorphous structures because the short duration of the pulses (20 µs) causes the faster supercooling and solidification of the formed mixed melt, which leads to the formation of larger amounts of amorphous and nanocrystalline structures and to an increased density and uniformity of coatings. From the EDS analysis of the presented results and our previous results [28,29,32], it was established that the main component of the glass-like zone from Figure 5b in the AlSi electrode coatings is Al, whose concentration can be changed within limits from 35 to 70–80% (Figure 8) by changing the pulse energy.

The results obtained in these studies create prerequisites for the targeted synthesis of increased amounts of certain phases in the surface layer by varying the modes and processing electrodes. For example, in RESD, with AlSi electrodes and the use a nitrogen environment and an appropriate selection of the duration and energy of the pulses (*E* = 0.04–0.07 J), TiN and TiCN can be synthesised in the formed layer, as can AlN or TiAlN, and various others can be purposefully synthesised in the formed layer [18,23,25,26,28]. In an air or oxygen environment, titanium oxides, Al_2_O_3_ and solid solutions of oxygen and nitrogen in titanium can be produced in the layer, and it is widely known that the oxide–nitride particles trapped in the titanium–aluminium matrix give higher hardness and wear resistance.

### 3.3. Tribological Characteristics of the Modified Surfaces

**Figure 9:**
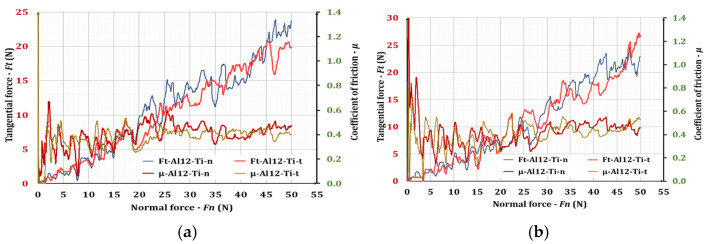
Coefficient of friction (μ) and tangential force (*F*_t_) of AlSi12-coated electrode on Ti-GR2 as a function of normal load 0–50 N. (**a**) ESD with AlSi12 electrode at *E* = 0.04 J; (**b**) ESD with AlSi12 electrode at *E* = 0.07 J.

Figure 9 shows the variation in the coefficient of friction, µ, and the tangential force, *F*_t_, depending on the normal load, *F*_n_, for ESD coatings from the AlSi12 electrode. With “n” and “t”, denoted tare he values of *F*_t_ and µ in two mutually perpendicular directions of movement of the diamond indenter relative to the coating.

It can be seen (Figure 9) that the coefficient of friction of the coated surfaces takes values of 0.4–0.55, which is close to those obtained in the work [32], and are smaller compared to those of the substrates (the original SLM titanium surfaces), which showed values around 0.6. The coefficient of friction values for the AlSi electrode coatings are 10–15% lower than those for the NW electrode coatings, which is consistent with the better uniformity and the lower surface roughness obtained with AlSi electrodes. It is also seen that the coefficient of friction of the coated surfaces with a pulse energy of 0.07 J is about 15% higher than that with an energy of 0.04 J, which corresponds to the lower values of roughness parameters at a lower pulse energy.

The influence of the normal load on the coefficient of friction of the coated surfaces is too weak, and the values for a normal load above 25 N are almost constant. The obtained coefficient of friction values are close and even lower than those received in the work in [33], where values of 0.76 and 0.57 were reported for titanium substrate and titanium substrate coated via ESD, respectively, in the case of a GCr15 ball slide at a sliding speed of 40 mm/s under a load of 5 N. Slight differences in the character of the graphs and the linear correlation between the coefficient of friction and friction force are clearly due to indenter oscillations caused by the specific surface relief and microroughness of the coatings. The fluctuations are higher for the coatings applied with a pulse energy of 0.07 J (Figure 9b), where the irregularities and roughness are higher.

**Figure 10:**
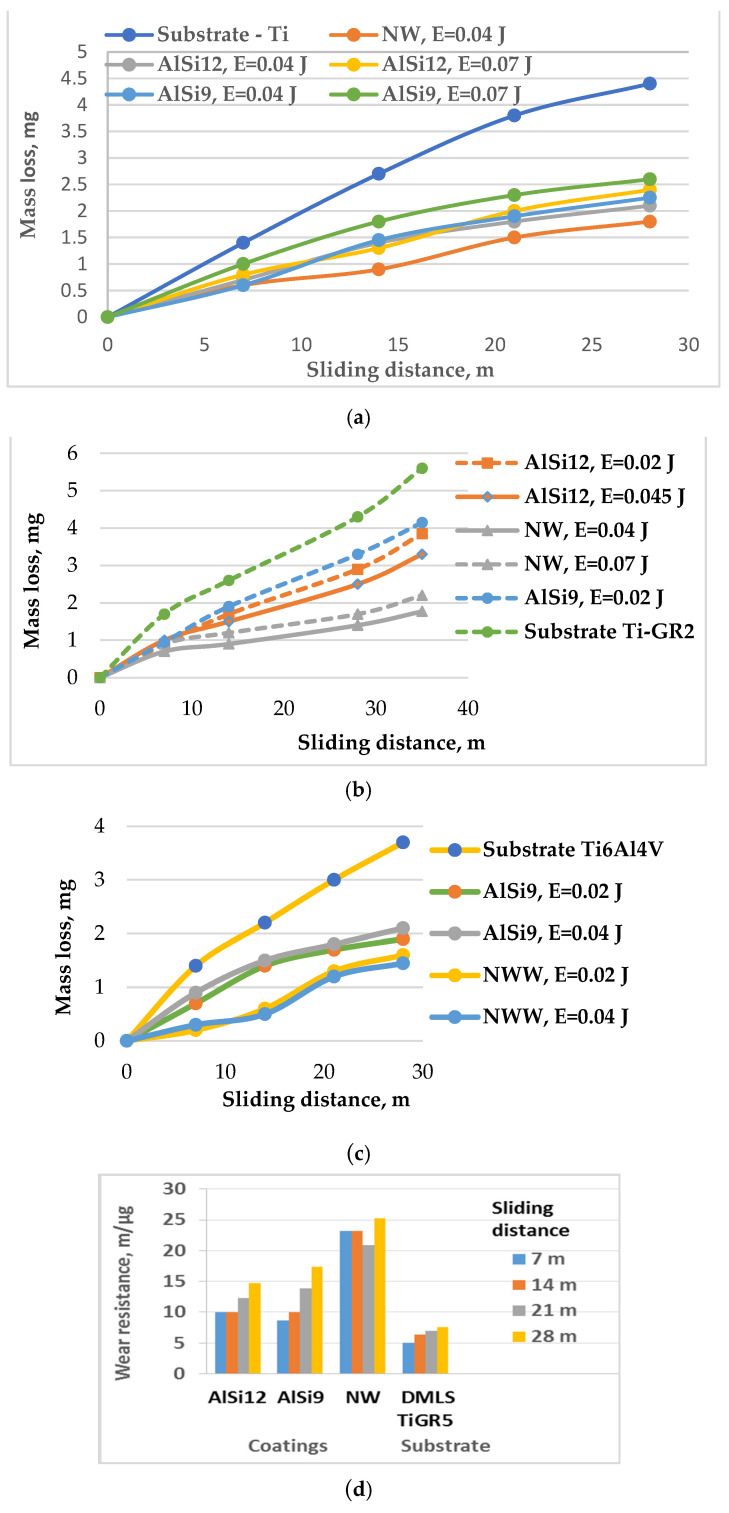
Wear and wear resistance against the sliding distance of the tested coatings on Ti-GR2 and Ti6Al4V (normal load of 5 N). (**a**) Mass loss of ESD coatings on SLM Ti-GR5 against the sliding distance; (**b**) Mass loss of LESD coatings on SLM Ti-GR5 against the sliding distance; (**c**) Mass loss of LESD coatings on DMLS Ti-GR5 against the sliding distance; (**d**) Wear resistance of LESD coatings on DLMS Ti-GR5 at a pulse energy of 0.04 J.

Figure 10 shows the development of mass loss and wear resistance as a function of sliding distance for RESD surfaces modified with the used electrodes at different pulse energy values.

Observations of the wear process give us reason to accept that abrasive wear and plastic deformation are dominant in the process. The development of wear starts with the breaking off of the coating particles, formed via transfer in a brittle (not fully melted) state, and also by the pores and microcracks in the coating. The detached hard abrasive particles together with the sandpaper particles participate in subsequent contact, contributing to an increase in the wear and its spread over the entire surface. From Figure 10, it can be found that the coatings show lower wear rates and, in addition to reducing surface defects, can be used to increase the durability of SLM titanium samples. The wear of the coatings applied with both electrodes was 2 to 3.5 times lower than that of the uncoated SLM samples.

The tribological tests showed that the smoother electrospark layers under RESD with a pulse energy of 0.03–0.04 J and the NW electrode had up to four times greater wear resistance than the uncoated ones did (Figure 10d). Their wear is up to 1.3 times less than that of the coated ones at an energy of 0.07 J. (Figure 10a–c). The samples coated with AlSi electrodes at pulse energy of 0.02–0.04 J show up to a 2.5-fold increase in wear resistance compared to that of the SLM samples. The lower wear of the samples modified with the NW electrode is explained by the presence of both carbides and borides, as well as the NiCrBSi solder mass, the components of which form more in number and a greater amount of new high-hardness and intermetal-lic phases than those of the AlSi electrodes. Similar results were obtained using NiCrBSi alloys and other surface treatment methods [34].

It is noted that the classic surface microgeometric indicators do not reflect the actual situation of sliding contact. As the pulse energy increases from 0.01 to 0.04 J, the roughness parameters (Figure 3a,b) also increase, and the wear resistance should decrease, but it increases. This increase in wear resistance is due to the increase in the content of hard, ultra-dispersed, and amorphous wear-resistant phases and the increased microhardness of the surface layer, as well as the formation of new intermetallic compounds and wear-resistant phases. On the other hand, the elimination of surface defects, the reduction in the coefficient of friction, and the formation of coatings with a specific surface relief are also favourable for wear resistance.

At a pulse energy of above 0.04–0.07 J, however, the influence of roughness on wear increases and the wear resistance shows a tendency to decrease, while at pulse energy of 0.07 J, it remains about 30% lower than the maximum value reported. It can be assumed that the most likely reason for the greater wear of the 0.07 J pulse energy of coated surfaces is their higher roughness parameters and the increased amount of electrode material carried over in the solid (incompletely melted) state.

When using AlSi electrodes, the decrease in wear resistance at the energy of 0.07 J is less pronounced, and the most complete erasure of the traces, defects and irregularities is observed, while the lowest roughness and at the same time a 1.5–2.8-fold higher hardness and wear resistance were obtained.

The analysis of the results led to the conclusion that through RESD with the used eutectic glass-like AlSi12 and the multi-component NW electrodes, prerequisites and possibilities were created for the purposeful formation of specific compositions and properties of the frictional contact surface. This allows, without using complex and expensive methods, us to carry out processing in such a way as to ensure both a reduction in roughness and the elimination of defects on SLM surfaces, as well as an increase in their wear resistance and the achievement of improved friction parameters.

## 4. Conclusions

The use of RESD with a low pulse energy (up to 0.07 J) and with low-melting AlSi electrodes on the surface of SLM and DMLS titanium alloys with different starting roughness allows the obtention of dense and uniform coatings with roughness and thickness values which, through the processing modes, can be changed to within *Ra* = 2–4 µm and δ = 6–15 µm. Coatings applied to surfaces with a roughness of *Ra* ≥ 6 μm allow a reduction in both the roughness and surface defects from 3D processing.

The technological solution proposed in this work creates prerequisites and opportunities for chemical exothermic reactions to occur between the electrode components, the inter-electrode medium and the substrate, which through the selection of suitable electrodes and pulse energy allow the formation of new wear-resistant compounds, intermetallic phases and amorphous crystalline structures in the composition of the modified surfaces.

The use of RESD with AlSi9 and AlSi12 electrodes allows a reduction in the roughness of SLM and DMLS titanium alloys by two to four times and allows the filling of the initial cracks and micropores. When using the hard alloy NW electrodes, the roughness of the AT surfaces was reduced by 2 to 2.8 times, and the elimination of defects was not complete. With an initial roughness of the titanium alloys of *Ra* = 6–12 µm, the lowest achieved roughness after RESD via LESD with AlSi electrodes and a single-pulse energy of *E* = 0.02–0.03 J was of the order of *Ra* = 1.8–2.2 µm.

The highest wear resistance is shown by the modified surfaces with the “NW” electrodes, and this is up to four times higher than that of the uncoated ones. The surfaces modified with AlSi electrodes, however, obtained the most complete erasure of traces, defects and irregularities, the lowest roughness, and at the same time, two- to three-fold higher hardness and wear resistance. The improvement in surface characteristics and tribological properties of 3D materials is achievable by using eutectic AlSi electrodes and the simple, economical and ecological RESD method with a pulse energy of 0.02–0.04 J.

The application of RESD after SLM and DMLS production processes provides the following possibilities: (i) a reduction in the defects and the roughness of the surfaces; (ii) the purposeful synthesis of new wear-resistant phases and amorphous-nanocrystalline surface structures and an increase in the wear resistance and scope of the application of titanium surfaces; (iii) an avoidance of the complex and expensive finishing processes after SLM and DMLS, expanding the application of the 3D printing of titanium products and reducing the cost of energy, labour and materials.

## Figures and Tables

**Table 1:** Used mode parameters and regimes.

Under LESD							Under ESD with Vibrating Electrode		
N	*I*, A	*T*_i_, μs	*C*, μF	*f*, kHz	*n*	*E*, J	N	*C*, μF	*E*, J
1	16	12	0.68	8	3	0.02	5	3.5	0.01
2	22.4	12	1	8	3	0.03	6	7	0.02
3	19.2	20	2.5	5	3	0.04	7	10	0.03
4	22.4	20	4.4	5	3	0.045	8	12	0.04
							9	20	0.07

**Table 2:** Roughness, (*Ra*, *Rq*, *Rz*, and *Rt*), thickness (*δ*) and microhardness (*HV*) of ESD and LESD coatings at 3 passes on DMLS and SLM substrates with different initial roughness values.

№	Electrode/Coating	*Ra*, µm	*Rq*, µm	*Rz*, µm	*Rt*, µm	*δ*, µm	*HV*, GPa
1	Substrate DMLS Ti-GR2	10.1	9.8	28.79	30.71	-	3.4
2	AlSi12, LESD, *E* = 0.02 J	2.49	2.64	7.07	7.14	8.8	7.87
3	AlSi12, LESD *E* = 0.045 J	2.76	3.02	7.81	7.88	13	9.47
4	AlSi12, ESD *E* = 0.04 J	3.42	3.87	15.54	22.3	14	8.85
5	AlSi9, ESD *E* = 0.04 J	2.67	3.14	14.1	19.7	12	8.63
6	NW, ESD *E* = 0.04 J	4.27	5.23	15.4	22.9	14	11.87
7	Substrate, SLM Ti-GR5	12.5	13.44	35.51	37.5	-	3.75
8	NW, LESD, *E* = 0.045 J	3.58	4.63	9.27	10.59	12	11.56
9	NW, ESD, *E* = 0.07 J	4.94	5.85	16.18	23.85	16	12.34
10	AlSi12, LESD, *E* = 0.045 J	3.65	4.25	11.1	14.3	14	9.84
11	AlSi9, LESD, *E* = 0.045 J	3.22	4.85	11.54	13.85	12	8.54

## Data Availability

Data are contained within the article.
